# Protein phosphatase 2A regulates xanthine oxidase-derived ROS production in macrophages and influx of inflammatory monocytes in a murine gout model

**DOI:** 10.3389/fphar.2022.1033520

**Published:** 2022-11-17

**Authors:** Sandy Elsayed, Khaled A. Elsaid

**Affiliations:** Department of Biomedical and Pharmaceutical Sciences, Chapman University School of Pharmacy, Irvine, CA, United States

**Keywords:** protein phosphatase 2a, xanthine oxidase, gout, macrophages, fingolimod (FTY-720)

## Abstract

**Background:** Gout is a common arthritis, due to deposition of monosodium urate (MSU) crystals which results in IL-1β secretion by tissue-resident macrophages. Xanthine oxidase (XO) catalyzes uric acid (UA) production and in the process, reactive oxygen species (ROS) are generated which contributes to NLRP3 inflammasome activation. Protein phosphatase 2A (PP2A) may be involved in regulating inflammatory pathways in macrophages. The objective of this study was to investigate whether PP2A regulates gout inflammation, mediated by XO activity modulation. We studied UA and ROS generations in MSU stimulated murine bone marrow derived macrophages (BMDMs) in response to fingolimod phosphate, a PP2A activator, and compared its anti-inflammatory efficacy to that of an XO inhibitor, febuxostat.

**Methods:** BMDMs were stimulated with MSU, GM-CSF/IL-1β or nigericin ± fingolimod (2.5 μM) or febuxostat (200 μM) and UA levels, ROS, XO, and PP2A activities, *Xdh (XO)* expression and secreted IL-1β levels were determined. PP2A activity and IL-1β in MSU stimulated BMDMs ± N-acetylcysteine (NAC) (10 μM) ± okadaic acid (a PP2A inhibitor) were also determined. M1 polarization of BMDMs in response to MSU ± fingolimod treatment was assessed by a combination of iNOS expression and multiplex cytokine assay. The *in vivo* efficacy of fingolimod was assessed in a murine peritoneal model of acute gout where peritoneal lavages were studied for pro-inflammatory classical monocytes (CMs), anti-inflammatory nonclassical monocytes (NCMs) and neutrophils by flow cytometry and IL-1β by ELISA.

**Results:** Fingolimod reduced intracellular and secreted UA levels (*p < 0.05*), *Xdh* expression (*p < 0.001*), XO activity (*p < 0.001*), ROS generation (*p < 0.0001*) and IL-1β secretion (*p < 0.0001*), whereas febuxostat enhanced PP2A activity (*p < 0.05*). NAC treatment enhanced PP2A activity and reduced XO activity and PP2A restoration mediated NAC’s efficacy as co-treatment with okadaic acid increased IL-1β secretion (*p < 0.05*). Nigericin activated caspase-1 and reduced PP2A activity (*p < 0.001*) and fingolimod reduced caspase-1 activity in BMDMs (*p < 0.001*). Fingolimod reduced iNOS expression (*p < 0.0001*) and secretion of IL-6 and TNF-α (*p < 0.05*). Fingolimod reduced CMs (*p < 0.0001*), neutrophil (*p < 0.001*) and IL-1β (*p < 0.05*) lavage levels while increasing NCMs (*p < 0.001*).

**Conclusion:** Macrophage PP2A is inactivated in acute gout by ROS and a PP2A activator exhibited a broad anti-inflammatory effect in acute gout *in vitro* and *in vivo*.

## Introduction

Gout is a common form of crystal induced arthropathy, characterized by acute episodes of pain and inflammation in affected joints (Busso and So. 2010; Taylor et al., 2015; Elfishawi et al., 2018; Chen-Xu et al., 2019; Dalbeth et al., 2019). The causative factor in gouty arthritis is the deposition of poorly soluble monosodium urate monohydrate crystals in periarticular tissues which in turns triggers an innate immune response mediated by tissue-resident macrophages (Dalbeth et al., 2019). As a product of purine base metabolism, uric acid (UA) is predominantly excreted by the kidneys and in the setting of chronic kidney insufficiency, UA serum level increases and when its saturation concentration is exceeded, UA may precipitate in the form of needle-shaped crystals (Martillo et al., 2014; Pascual et al., 2015). The accepted pathogenic pathway of gout involves the phagocytosis of urate crystals by macrophages, mediated by receptors e.g., the toll-like receptors (TLRs) and CD44 (Liu-Bryan et al., 2005; Bousoik et al., 2020). Following their uptake, urate crystals activate the nucleotide-binding oligomerization domain-like receptor family, pyrin domain-containing 3 (NLRP3) inflammasome, a multiprotein complex of the NLRP3 protein, ASC protein, and procaspase-1 enzyme (Swanson et al., 2019). Procaspase-1 is subsequently converted to active caspase-1, leading to the processing and secretion of the pro-inflammatory cytokine, interleukin-1 beta (IL-1β) (Swanson et al., 2019).

The NLRP3 inflammasome can be activated by various stimuli including pathogen and damage associated molecular patterns, insoluble factors e.g., urate and cholesterol crystals, soluble factors including nigericin and ATP, and by changes in cytosolic levels of K^+^ and Ca^2+^ (Martinon et al., 2006; Cruz et al., 2007; Freigang et al., 2011). Our understanding of the molecular pathways that mediate NLRP3 inflammasome activation in response to these stimuli remains limited. Recently, the activity of xanthine oxidase (XO) was implicated in the activation of NLRP3 inflammasome and IL-1β release in response to insoluble crystals (Ives et al., 2015). XO is the oxidized form of xanthine oxidoreductase, and a significant source of cellular reactive oxygen species (ROS) as it produces superoxide and hydrogen peroxide in addition to UA, and its activity was shown to increase during immune activation, where it potentially mediates TLR-dependent inflammatory arthritis (Kim et al., 2014). ROS but not UA was shown to mediate NLRP3 inflammasome activation in urate crystal stimulated macrophages, and inhibition of XO activity by febuxostat reduced cellular ROS, and inflammasome-dependent IL-1β processing (Ives et al., 2015).

Protein phosphatase 2A (PP2A) is a highly conserved serine/threonine phosphatase enzyme that is a heterotrimer composed of catalytic, structural, and regulatory subunits, and plays an important role in regulating various signal transduction pathways (Schuhmacher et al., 2019). Dysfunction in cellular PP2A activity level is associated with neurodegenerative diseases, diabetes, heart disease and cancers (Schuhmacher et al., 2019). PP2A is also an important regulator of the innate immune response by macrophages, where conditional knockdown of PP2A catalytic subunit enhanced tumor necrosis factor alpha (TNF-α) expression in LPS stimulated macrophages (Sun et al., 2017). In the context of gout, we have previously shown that PP2A knockdown in human THP-1 monocytes exacerbated IL-1β release in response to urate crystal stimulation (Qadri et al., 2021a). In addition, PP2A activity was reduced in human monocytes in response to urate crystals and restoration of PP2A activity by fingolimod, a PP2A activator, suppressed IL-1β expression and release by THP-1 monocytes (Qadri et al., 2021a).

The objective of this study was to investigate whether the role of PP2A in regulating gout inflammation is mediated by XO activity modulation. We studied ROS and UA generations in urate crystal stimulated murine bone marrow derived macrophages (BMDMs) in response to fingolimod phosphate treatment and compared its anti-inflammatory efficacy to that of an XO inhibitor, febuxostat. Furthermore, we studied the role of ROS in regulating PP2A activity and investigated the *in vivo* efficacy of fingolimod phosphate in a murine peritoneal model of acute gout. We hypothesized that PP2A activation reduces XO-derived ROS generation and downstream IL-1β processing in urate crystal stimulated BMDMs.

## Materials and methods

### Preparation of macrophage serum-free and complete media

Dulbecco’s Modified Eagle Medium/Nutrient Mixture F-12 (DMEM/F-12, GlutaMAX^TM^ supplement) (ThermoFisher Scientific) was supplemented with 1% Penicillin/Streptomycin (Sigma Aldrich) ± 10% Fetal Bovine Serum (FBS) (ATCC), and 20 ng/ml recombinant murine macrophage colony-stimulating factor (M-CSF) (R&D Systems).

### Culture of bone marrow progenitor cells and generation of BMDMs

Harvest of murine bone marrows was performed as previous described, using 2–3 months-old male C57BL/6 mice (JAX) (Zhang et al., 2008; Qadri et al., 2018). Harvest of murine bone marrows was approved by the IACUC committee at Chapman University. Cells were centrifuged at 400x*g*, 4°C for 10 min. The supernatant was discarded, and cells were resuspended and seeded in T75 flasks at ∼4.0 × 10^6^/mL in 10 ml pre-warmed complete media and incubated at 37°C in 5% CO_2_ incubator. On days 3 and 5, the supernatant was discarded, and cells were washed with 4 ml Dulbecco’s Phosphate Buffered Saline (DPBS) (Sigma Aldrich). On day 7, the supernatant was discarded, and BMDMs were incubated with cell stripper nonenzymatic cell dissociation solution (Sigma Aldrich) at 37°C for 5 min and cells were gently scraped. An equal volume of complete media was added, and stripped cells were centrifuged at 400x*g*, 4°C for 10 min. The supernatant was discarded, and macrophages were resuspended in complete media.

### Priming and activation of BMDMs through crystal and non-crystal pathways

BMDMs were seeded in tissue culture (TC)-treated 6-well plates (1.0 × 10^6^ cells/well) and left to adhere for 24 h. The supernatant was removed, and macrophages were washed twice with DPBS then Pam3CSK4, a toll-like receptor 2 agonist (100 ng/ml; Invivogen) (Feng et al., 2019), was added in serum-free media and incubated for 24 h. Cells were pretreated with 2.5 μM fingolimod phosphate (Cayman) or 200 μM febuxostat (Sigma-Aldrich) for 3 h. Finally, macrophages were stimulated with pyrogen-free monosodium urate (MSU) crystals (250 μg/ml: Invivogen) for 3 h. This results in a total incubation time of 6 h with fingolimod or febuxostat. The rationale for selecting 6 h of drug incubation was based on our previous observation that maximal PP2A activation by fingolimod in human THP-1 monocytes was at 6 h (Qadri et al., 2021a). The fingolimod dose selection was based on maximal PP2A activation (Qadri et al., 2021a), and febuxostat’s dose was the dose that was shown to inhibit XO activity and IL-1β secretion in murine BMDMs (Ives et al., 2015). Similarly, for non-crystal induced BMDM activation, BMDMs were primed with GM-CSF (20 ng/ml; R&D Systems) for 24 h then stimulated with recombinant murine IL-1β (1 ng/ml) (R&D Systems) for 6 h. The rationale for incorporating GM-CSF/IL-1β stimulation was that both cytokines are elevated in gout (Shaw et al., 2014) making the stimulation biologically relevant and allowing us to investigate UA production by XO in macrophages in the absence of urate crystals. Supernatants were collected and stored at -20°C. Cell isolates were collected, and total protein contents were quantified using Pierce^TM^ BCA Protein Assay Kit (Thermo Fisher Scientific) according to manufacturer’s instructions.

### Intracellular and secreted UA, xanthine dehydrogenase/XO expression and activity in BMDMs

We studied the generation of UA by BMDMs *via* assessment of intracellular UA levels in cell lysates following priming BMDMs with Pam3CSK4 for 24 h and treatment with MSU crystals (250 μg/ml) ± fingolimod phosphate (2.5 μM) or febuxostat (200 μM) and normalization to cell lysate total protein using UA assay kit (Sigma Aldrich). In addition, we measured secreted UA in GM-CSF/IL-1β stimulated BMDMs. We also quantified intracellular XO activity in BMDMs following MSU crystals or GM-CSF/IL-1β stimulations and normalized to cell lysate total protein, using a commercial XO activity assay kit (Sigma Aldrich). Xanthine dehydrogenase (Xdh) gene expression was performed following total RNA extraction, quantification, cDNA synthesis and quantitative PCR (qPCR) as previously described (Qadri et al., 2018), using commercially available primers (Mm00442110_m1) (ThermoFisher Scientific). The cycle threshold (Ct) value of Xdh was normalized to the Ct value of GAPDH (Mm99999915_g1) in the same sample, and relative expression was calculated using the 2^−ΔΔCT^ method (Livak and Schmittgen, 2001).

### PP2A activity and IL-1β production by BMDMs

PP2A was immunoprecipitated from BMDM cell lysates following Pam3CSK4 priming for 24 h ± fingolimod phosphate (2.5 μM) or febuxostat (200 μM) for 3h, and stimulation with MSU crystals (250 μg/ml) for 3 h. PP2A activity was determined as previously described (Qadri et al., 2021a) and normalized to total protein in cell lysate (Emdmillipore). Secreted IL-1β levels were determined by ELISA (R&D Systems).

### ROS quantification in BMDMs

BMDMs were seeded in 96-well plate (50,000 cells/well) for 24 h. Cells were primed with Pam3CSK4 for 24 h then incubated with either fingolimod phosphate (2.5 μM), febuxostat (200 μM) or NAC (10 μM) for 3 h. Finally, cells were stimulated with MSU crystals (250 μg/ml) for 3 h. ROS generation was measured using DCFDA/H2DCFDA cellular ROS assay (Abcam) according to manufacturer’s instructions.

### *Nrf2* expression and immunostaining in MSU challenged murine macrophages

BMDMs were seeded in TC-treated 6-well plates (1.0 × 10^6^ cells/well) then primed with 100 ng/ml Pam3CSK4 for 24 h. Cells were treated as described above. Total RNA extraction, quantification, cDNA synthesis and qPCR were performed, using commercially available primers for factor erythroid 2-related factor 2 (Nrf-2) (Mm00477784_m1) (Thermo Fisher Scientific), and relative expression was determined as above. In a separate set of experiments, murine J774 macrophages (ATCC) were cultured in DMEM media +1% Penicillin/Streptomycin (Sigma Aldrich). Assessment of Nrf2 protein was performed using immunofluorescence and determination of corrected total cell fluorescence (CTCF) using a Nikon Ti-E Confocal laser microscope. J774 macrophages (400,000 cells/well) were cultured on collagen type-I-coated 22 mm glass coverslips for 24 h in complete growth medium. Priming was done with 100 ng/ml Pam3CSK4 in serum-free media for 24 h. Cells were pretreated with 2.5 μM fingolimod phosphate or 200 μM febuxostat for 3 h. Finally, macrophages were stimulated with MSU crystals (250 μg/ml) for 3 h. Subsequently, cells were fixed in 10% neutral buffered formalin for 15 min and washed twice with PBS. Cells were permeabilized using 0.02% Triton X-100 in PBS and blocked using 2% BSA (Sigma-Aldrich) in PBS for 2 h at room temperature. Probing for Nrf2 was performed using anti-Nrf2 rabbit monoclonal antibody (1:1,000; Cell Signaling) overnight at 4°C. Secondary probing was done with Alexa Fluor 488-labeled goat anti-rabbit antibody (1:1,000; Abcam) for 1 h at 4°C. Following washing with PBS, cells were mounted on DAPI mounting shield (Abcam) and CTCF was quantified across groups and normalized to control values.

### M1 and M2 markers’ expressions and cytokine array assays in response to fingolimod treatment

BMDMs were seeded in TC-treated 6-well plates (1.0 × 10^6^ cells/well) then primed with 100 ng/ml Pam3CSK4 for 24 h. Cells were treated as described above. Genes of interest included IL-1β (Mm00434228_m1), inducible nitric oxide synthase (iNOS) (Mm00440502_m1), chemokine CCL2 (Mm00441242_m1), interleukin-10 (IL-10) (Mm01288386_m1), and arginase (Arg) (Mm00475988_m1). All primers and probes are commercially available (ThermoFisher Scientific). The cycle threshold (Ct) values of genes of interest were normalized to the Ct value of GAPDH (Mm99999915_g1) in the same sample, and relative expression was determined as above. Media supernatants were analyzed for colony stimulating factors (CSF), cytokines and chemokines using the proteome profiler mouse cytokine array kit, Panel A (R&D Systems).

### ROS generation, PP2A and caspase-1 activities and IL-1β secretion in nigericin-stimulated BMDMs and impact of fingolimod treatment

BMDMs were seeded as described above and treated with Pam3CSK4 for 24 h followed by fingolimod and febuxostat treatments for 3 h followed by nigericin (5 μM; Invivogen) for 3 h. ROS generation, PP2A activity and IL-1β levels were determined as described above. In a separate set of experiments, BMDMs (50,000 cells per well) were seeded in 96 well plates and stimulation and treatments were conducted as described above and caspase-1 activity was measured using Caspase 1 (active) Staining Kit (Abcam).

### *In vivo* murine peritoneal model of acute gout and impact of fingolimod treatment on influx of monocytes and neutrophils and peritoneal lavage IL-1β levels

We employed the murine peritoneal model of acute gout as previously described (Elsayed et al., 2021). Our experiments were approved by the IACUC committee at Chapman University. A total of 25 male mice (2–3 months-old) (C57BL/6; Jax) were utilized and randomly assigned to control (n = 5), MSU + vehicle (n = 8), MSU + fingolimod (n = 8) and fingolimod only (n = 4). Pyrogen-free MSU crystals (2 mg in 200 μL), vehicle (200 μL) or fingolimod (5 mg/kg; 200 μL) were administered intraperitoneally and peritoneal lavaging was performed at 4 h. Lavaging was performed by injecting 3 ml of cold DPBS into the peritoneal cavity followed by shaking for 30–60 s and lavage aspiration. Lavage fluids were centrifuged at 3,000 rpm for 5 min at 4°C, and cell pellets were resuspended in PBS and immunophenotyping of CMs, NCMs and neutrophils was conducted as previously described (Elsayed et al., 2021). CMs were identified as CD11b+ Ly6C^hi^ CCR2+, while NCMs were identified as CD11b+ Ly6C^lo^ CD43^hi^ CX3CR1+ and neutrophils were identified as Ly6G + Ly6B.2+ (Elsayed et al., 2021). We have previously shown the detailed gating strategies for murine CMs, NCMs and neutrophils in peritoneal lavage cell pellets (Elsayed et al., 2021). In summary, singlets were initially identified followed by gating for viable cells using Zombie violet dye. CD11b+ cells were identified and two populations: Ly6C^hi^ and Ly6C^lo^ cells were gated. We subsequently gated for CCR2 positivity in the Ly6C^hi^ population to identify CMs and gated for CD43 and CX3CR1 in the Ly6C^lo^ population to identify NCMs (Elsayed et al., 2021). For neutrophils, singlet viable cells were gated for Ly6G and Ly6B.2 markers. We utilized commercially available fluorochrome-conjugated antibodies as described and positivity thresholds were set using fluorescence minus one (FMO) control (Elsayed et al., 2021). Intracellular staining for XO was performed using an intracellular staining protocol as described (Qadri et al., 2021b) and a primary rabbit anti-xanthine oxidase antibody (1:100) and a secondary goat anti-rabbit IgG-Alexa Fluor 488 (1:100) (Abcam). Immunophenotyping for CMs and NCMs was performed independently of neutrophil immunophenotyping, and cells in populations of interest were quantified using Precision Counting Beads (BioLegend). Lavage IL-1β levels were determined by ELISA (R&D Systems).

### Statistical analyses

Statistical analyses were conducted as described (Qadri et al., 2021a). We utilized one-way analysis of variance (ANOVA) followed by Tukey’s *post*-hoc test. A *p* value of <0.05 was considered statistically significant. Data are represented graphically as scatter plot bar graphs with mean ± standard deviation indicated. In our *in vitro* BMDM experiments, each data point represents an independent experiment conducted with BMDMs derived from 2-3 animals. In our *in vivo* experiments, each data point represents an individual mouse.

## Results

### Fingolimod, a PP2A activator, reduced UA generation, XO expression and activity in macrophages and XO inhibition restored PP2A activity in urate crystal stimulated macrophages

**FIGURE 1:**
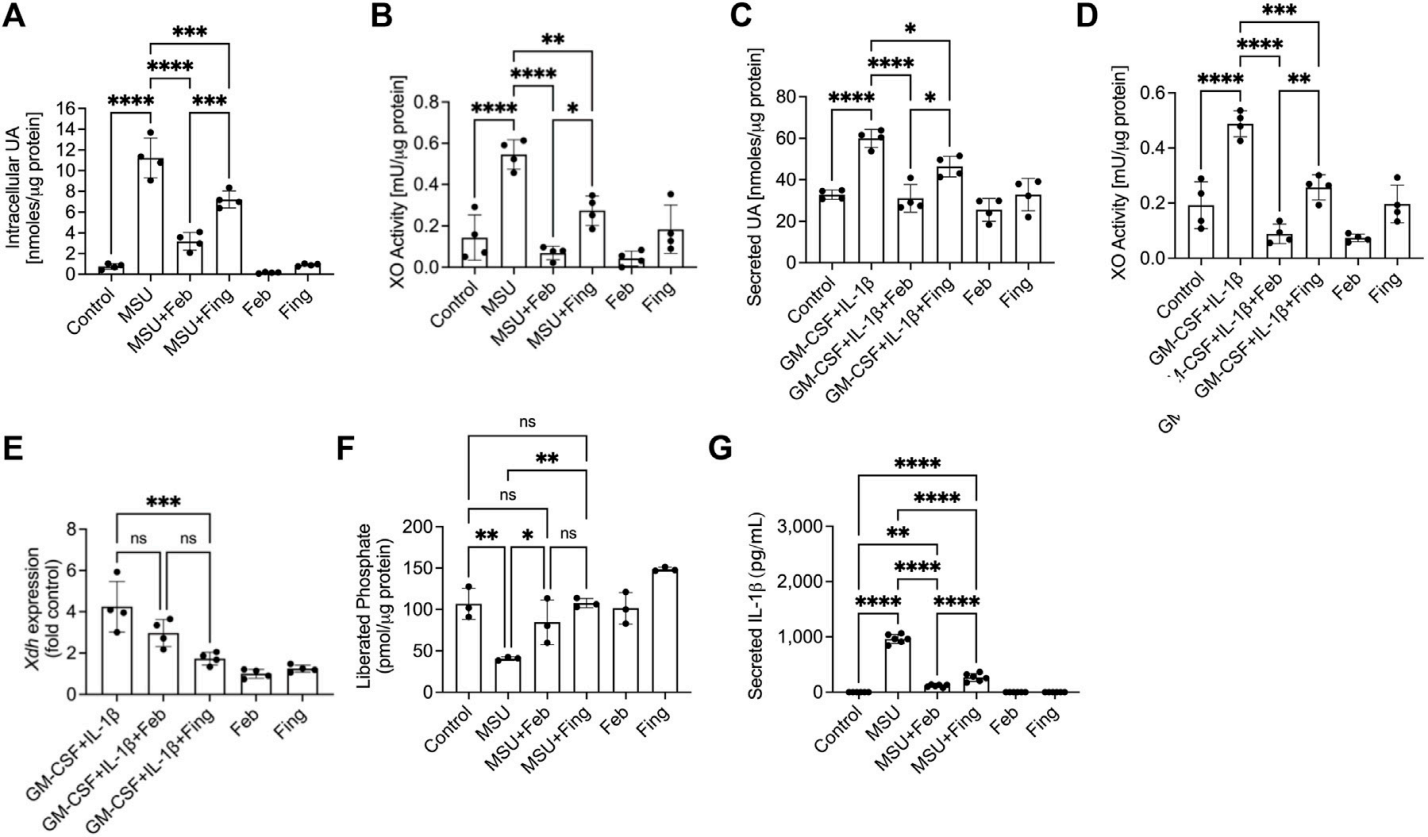
Regulation of uric acid (UA) production, xanthine oxidase (XO) and protein phosphatase 2A (PP2A) activities, XO (xanthine dehydrogenase; *Xdh*) expression and interleukin-1 beta (IL-1β) secretion by murine bone marrow derived macrophages (BMDMs) in response to Pam3CSK4 (TLR2 ligand) and monosodium urate (MSU) crystals or GM-CSF/IL-1β stimulations. BMDMs were primed with Pam3CSK4 (100 ng/ml) for 24 h. BMDMs were then incubated with febuxostat (Feb; 200 μM) or fingolimod (Fing; 2.5 μM) for 3 h followed by MSU crystals (250 μg/ml), and all assays were performed at 3 h. Alternatively, BMDMs were primed with GM-CSF (20 ng/ml) for 24 h followed by IL-1β (1 ng/ml) ± drug treatments for 3 h. Data points represent independent experiments with two technical replicates per group. In each experiment, BMDMs were generated from the bone marrows of 2-3 animals. Control group represents untreated BMDMs. Statistical analysis was performed by ANOVA. ns: non-significant; *p < 0.05; **p < 0.01; ***p < 0.001; ****p < 0.0001. **(A)** Fingolimod reduced intracellular UA levels in MSU stimulated BMDMs. Febuxostat reduced intracellular UA levels to a greater extent to fingolimod. **(B)** Fingolimod reduced XO activity in MSU crystal stimulated BMDMs. Febuxostat reduced XO activity to a similar extent to fingolimod. **(C)** Fingolimod reduced secreted UA levels in GM-CSF/IL-1β stimulated BMDMs. Febuxostat reduced secreted UA levels to a greater extent to fingolimod. **(D)** Fingolimod reduced XO activity in GM-CSF/IL-1β stimulated BMDMs. Febuxostat reduced XO activity to a greater extent to fingolimod. **(E)** Fingolimod reduced Xdh expression in GM-CSF/IL-1β stimulated BMDMs. Febuxostat did not alter Xdh expression under the same conditions. **(F)** Fingolimod and febuxostat enhanced PP2A activity in MSU stimulated BMDMs. **(G)** Fingolimod reduced secreted IL-1β levels in MSU stimulated BMDMs. Febuxostat reduced IL-1β secretion to a greater extent to fingolimod.

To evaluate the impact of PP2A activation on XO activity and downstream UA production, we stimulated BMDMs with MSU crystals or with GM-CSF/IL-1β separately and observed that fingolimod reduced intracellular UA (*p < 0.001*; Figure 1A) and secreted UA (*p < 0.05*; Figure 1C) levels, *respectively*. Both MSU crystals (*p < 0.0001*; Figure 1B) and GM-CSF/IL-1β (*p < 0.0001*; Figure 1D) induced XO activity in BMDMs. Fingolimod suppressed XO activation in response to MSU crystals and GM-CSF/IL-1β (*p < 0.001* for both comparisons), whereas febuxostat expectedly reduced XO activity and UA production in our *in vitro* models (*p < 0.0001* across all comparisons). Fingolimod treatment reduced *Xdh* expression in GM-CSF/IL-1β stimulated BMDMs (*p < 0.001*; Figure 1E), while febuxostat treatment did not modify *Xdh* expression under the same conditions (*p > 0.05*). XO inhibition by febuxostat in MSU stimulated BMDMs enhanced PP2A activity (*p < 0.05*) similar to fingolimod, a PP2A activator (Figure 1F). Fingolimod exhibited an anti-inflammatory effect as evidenced by reducing IL-1β secretion by BMDMs (*p < 0.0001*; Figure 1G), wherein the secreted IL-1β level seen with febuxostat was lower than fingolimod (*p < 0.0001*). Our data suggest that fingolimod and febuxostat may share a common mechanistic pathway, specifically *via* enhancing PP2A activity resulting in downstream inhibition of IL-1β production. In addition, fingolimod appears to regulate XO enzymatic activity as a result of downregulating *Xdh* expression.

### ROS generation in MSU challenged BMDMs reduced PP2A activity and fingolimod treatment suppressed ROS generation with an associated enhancement in *Nrf2* expression

**FIGURE 2:**
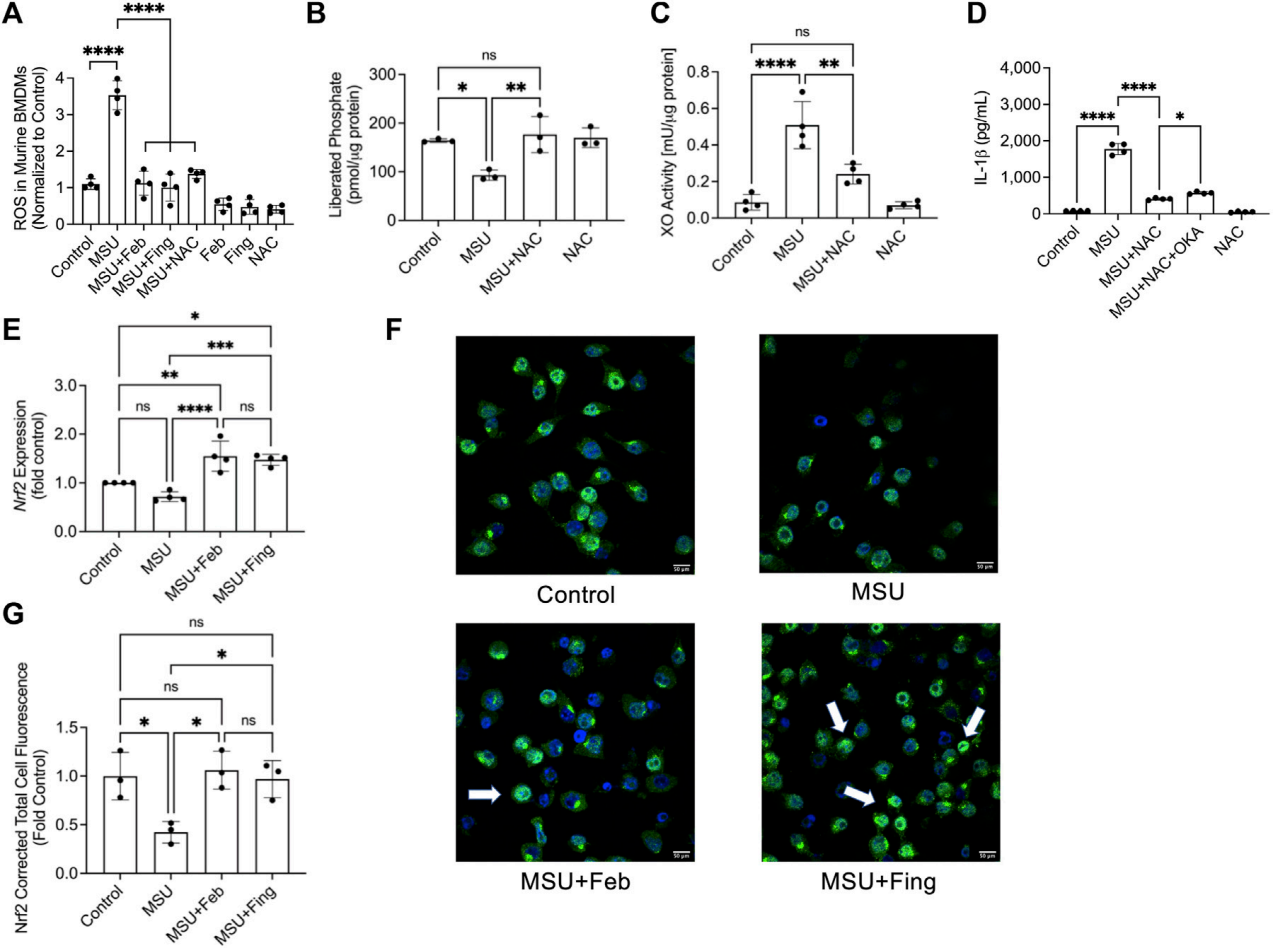
Urate crystal induced generation of reactive oxygen species (ROS) and its relationship to protein phosphatase 2A (PP2A) and xanthine oxidase (XO) activities, and nuclear factor erythroid 2-related factor 2 (Nrf2) expression in response to PP2A activation or XO inhibition in murine macrophages. Murine bone marrow derived macrophages (BMDMs) were primed with Pam3CSK4 (100 ng/ml) for 24 h. BMDMs were then incubated with febuxostat (February; 200 μM) or fingolimod (Fing; 2.5 μM) for 3 h followed by monosodium urate (MSU) crystals (250 μg/ml), and all assays were performed at 3 h. N-acetylcysteine (NAC) and okadaic acid (OKA) treatments were performed at 10 μM and 2.5nM, respectively. Nrf2 expression was quantified at the gene level by qPCR and at the protein level using immunofluorescence and estimating corrected total cell fluorescence (CTCF), normalized to control values. Data points represent independent experiments with two technical replicates per group. In each experiment, BMDMs were generated from the bone marrows of 2-3 animals. In the immunofluorescence studies, data points represent independent experiments with one technical replicate per group using murine J774 macrophage cell line. Control group represents untreated BMDMs or J774 macrophages. Statistical analysis was performed by ANOVA. ns, non-significant; *p < 0.05; **p < 0.01; ***p < 0.001; ****p < 0.0001. **(A)** Fingolimod reduced ROS generation in MSU stimulated BMDMs. Febuxostat and NAC reduced ROS generation to a similar extent to fingolimod. **(B)** NAC enhanced PP2A activity in MSU stimulated BMDMs. **(C)** NAC reduced XO activity in MSU stimulated BMDMs. **(D)** NAC reduced IL-1β secretion by MSU stimulated BMDMs, and OKA co-treatment attenuated that effect. **(E)** Nrf2 expression in BMDMs was enhanced with fingolimod and febuxostat treatment. **(F)** Representative confocal immunofluorescence images showing enhanced Nrf2 staining in MSU crystal stimulated murine J774 macrophages treated with fingolimod or febuxostat (as shown by arrows). **(G)** Fingolimod and febuxostat treatments enhanced Nrf2 cellular levels in MSU crystal stimulated J774 macrophages.

Exposure to MSU crystals generated ROS in BMDMs (*p < 0.0001*; Figure 2A). Accumulation of ROS was principally mediated by XO activation as febuxostat treatment suppressed ROS generation in BMDMs (*p < 0.0001*). PP2A activation by fingolimod also suppressed ROS generation (*p < 0.0001*). To further elucidate the relationship between XO-derived ROS and PP2A activity modulation, we studied the change in PP2A activity ± NAC. NAC treatment reduced ROS generation (*p < 0.0001*) in BMDMs with a downstream effect of recovering PP2A activity (*p < 0.01*; Figure 2B). Furthermore, NAC treatment reduced XO activity in MSU stimulated BMDMS (*p < 0.01*; Figure 2C). The causal effect of ROS accumulation on IL-1β secretion was further investigated when we treated MSU challenged BMDMs with NAC and observed that NAC treatment reduced IL-1β secretion (*p < 0.0001*; Figure 2D). Our data suggest that XO-derived ROS inactivated PP2A and the anti-inflammatory effect of the antioxidant, NAC might be mediated by restoring PP2A activity, and related inhibition of XO activity. To determine the extent to which PP2A activity level mediated NAC’s efficacy, we treated MSU challenged BMDMs with NAC ± okadaic acid (2.5 nM), a PP2A inhibitor. We observed that okadaic acid co-treatment attenuated NAC’s effect (*p < 0.05*; Figure 2D). The reversal of NAC’s effect by PP2A inhibition was not complete, indicating that NAC’s effect was partially mediated by restoration of PP2A activity.

We also investigated *Nrf2* expression in BMDMs and observed that while there was a trend towards lower expression in response to combined TLR2 agonist priming and urate crystal exposure, it did not reach statistical significance when compared to untreated control macrophages (*p > 0.05*; Figure 2E). Fingolimod enhanced *Nrf2* (*p < 0.001*) expression and this effect was also seen with febuxostat (*p < 0.0001*). There was no difference in the magnitude of *Nrf2* expression enhancement with both drug treatments (*p > 0.05*). We also investigated Nrf2 protein in a model of MSU crystal challenged murine macrophages using immunoprobing, and qualitatively observed more intense Nrf2 staining in fingolimod and febuxostat-treated cells (indicated by arrows; Figure 2F). Quantitatively, both drug treatments increased Nrf2 levels in murine macrophages (*p < 0.05* for both comparisons; Figure 2G). Our data suggest that 1) PP2A was inactivated in the setting of an inflammatory insult mediated by ROS accumulation, 2) PP2A activation suppressed inflammation *via* inhibition of XO-derived ROS generation and 3) fingolimod and febuxostat reduced ROS generation and enhanced *Nrf2* expression, an important transcriptional factor that plays a central role in regulating cellular antioxidant and cytoprotective genes.

### Nigericin reduced PP2A activity in BMDMs and fingolimod reduced nigericin-induced caspase-1 activity and IL-1β secretion

**FIGURE 3:**
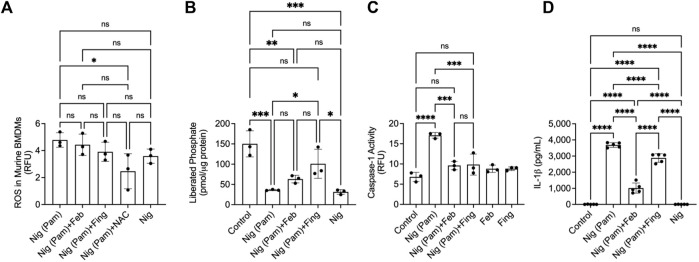
Protein phosphatase 2A (PP2A) activity in nigericin (Nig)-induced NLRP3 inflammasome activation and interleukin-1 beta (IL-1β) release by murine bone marrow derived macrophages (BMDMs) and efficacy of fingolimod, a PP2A activator, in regulating caspase-1 activation and IL-1β secretion. BMDMs were primed with Pam3CSK4 (100 ng/ml) for 24 h. BMDMs were then incubated with febuxostat (February; 200 μM), fingolimod (Fing; 2.5 μM), or N-acetylcysteine (NAC) (10 μM) for 3 h followed by Nig (5 μM), and all assays were performed at 3 h. Caspase-1 activity was determined fluorometrically, and data are presented as relative fluorescence units (RFU). Data points represent independent experiments with two technical replicates per group. In each experiment, BMDMs were generated from the bone marrows of 2-3 animals. Control group represents untreated BMDMs. Statistical analysis was performed by ANOVA. ns: non-significant; *p < 0.05; **p < 0.01; ***p < 0.001; ****p < 0.0001. **(A)** ROS in nigericin-treated BMDMs was reduced with NAC treatment. **(B)** Nigericin reduced PP2A activity in BMDMs. Fingolimod enhanced PP2A activity in nigericin-treated BMDMs. **(C)** Fingolimod reduced capsase-1 activity in nigericin-treated BMDMs. Febuxostat reduced caspase-1 activity to a similar extent to fingolimod. **(D)** Fingolimod reduced secreted IL-1β in nigericin-treated BMDMs. Febuxostat reduced IL-1β secretion to a greater extent to fingolimod.

Nigericin is an NLRP3 activator, by being a potassium ionophore and by inducing mitochondrial ROS generation. Fingolimod and febuxostat treatments did not alter ROS levels in Pam3CSK4+nigericin-treated BMDMs (*p > 0.05;* Figure 3A), while the positive control, NAC reduced ROS levels (*p < 0.05*). Nigericin treatment reduced PP2A activity in BMDMs (*p < 0.001*; Figure 3B), while fingolimod enhanced PP2A activity (*p < 0.05*) in nigericin-treated cells. Nigericin activated caspase-1 in BMDMs (*p < 0.0001*; Figure 3C) and fingolimod treatment reduced caspase-1 activity in this setting (*p < 0.001*). Also, fingolimod was efficacious in reducing IL-1β secretion by nigericin-stimulated BMDMs (*p < 0.0001*; Figure 3D). Similar to fingolimod, febuxostat suppressed caspase-1 activity (*p < 0.001*) and IL-1β secretion (*p < 0.0001*). Our data suggest that PP2A inactivation is evident in the pathways leading to NLRP inflammasome activation by nigericin and by insoluble crystals. In addition, PP2A activation reduced caspase-1 activity and IL-1β production in response to nigericin treatment.

### Fingolimod reduced the expression of M1 markers; iNOS, IL-1β, and CCL2 and the secretion of M-CSF, IL-1, IL-6 and TNF-α in MSU stimulated BMDMs

**FIGURE 4:**
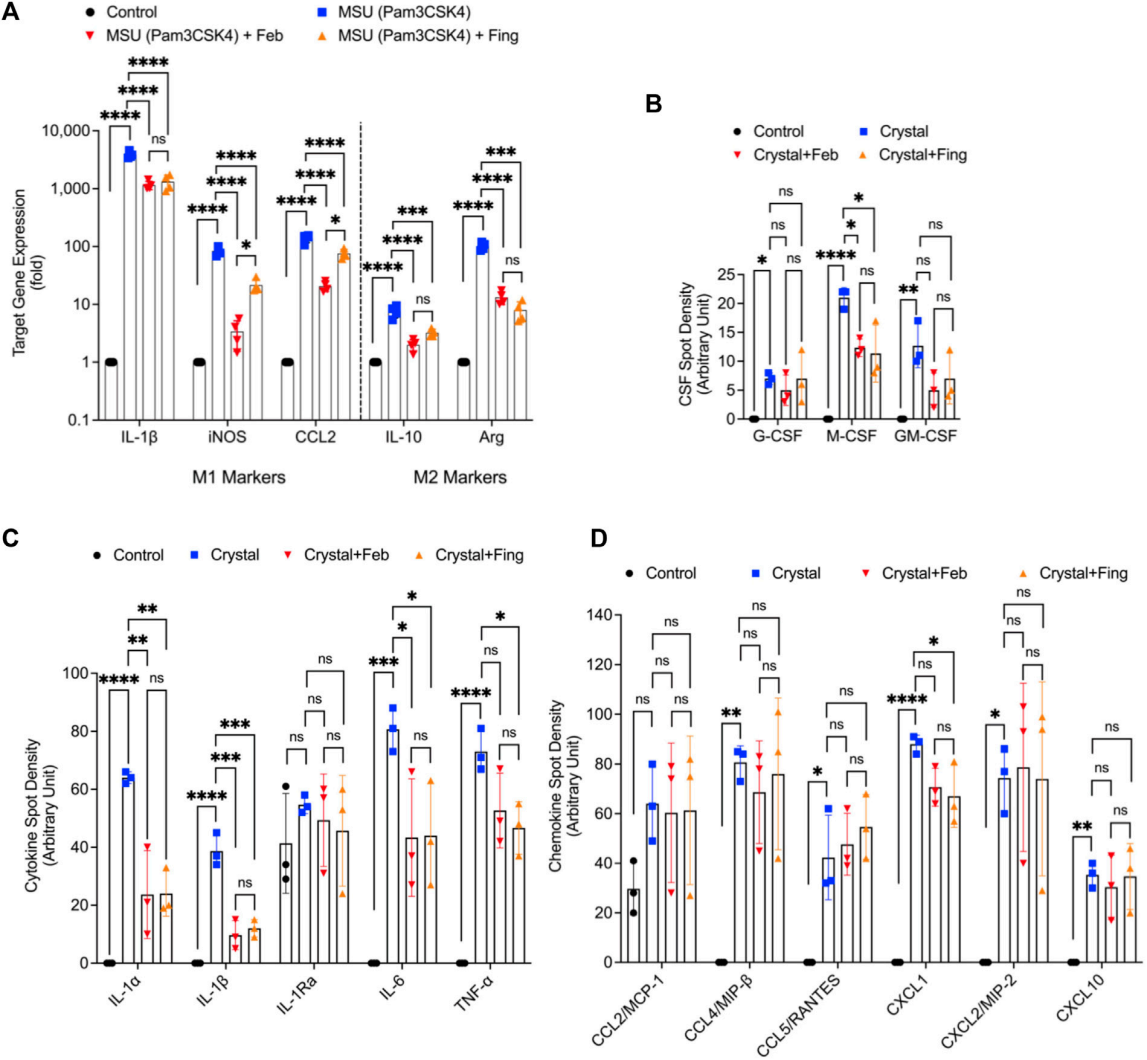
Regulation of M1 and M2 markers and profiling of cytokines and chemokines in monosodium urate (MSU) crystal challenged murine bone marrow derived macrophages (BMDMs) and impact of fingolimod and febuxostat treatments. BMDMs were primed with Pam3CSK4 (100 ng/ml) for 24 h. BMDMs were then incubated with febuxostat (February; 200 μM) or fingolimod (Fing; 2.5 μM) for 3 h followed by monosodium urate (MSU) crystals (250 μg/ml), and all assays were performed at 3 h. M1 markers’ gene expression included interleukin-1 beta (IL-1β), inducible nitric oxide synthase (iNOS), CCL2 and M2 markers; IL-10 and arginase-1 (Arg). Colony stimulating factor (CSF), cytokine and chemokine profiling was performed using a cytokine array panel. Data points represent independent experiments with two technical replicates per group. In each experiment, BMDMs were generated from the bone marrows of 2-3 animals. Control group represents untreated BMDMs. Statistical analysis was performed by ANOVA. ns: non-significant; *p < 0.05; **p < 0.01; ***p < 0.001; ****p < 0.0001. **(A)** Fingolimod and febuxostat reduced M1 markers’ expressions but did not increase M2 markers’ expressions. **(B)** Fingolimod and febuxostat reduced M-CSF secretion by MSU stimulated BMDMs. **(C)** Fingolimod and febuxostat reduced IL-1α, IL-1β, IL-6 and TNF-α secretions by MSU stimulated BMDMs. **(D)** Fingolimod reduced CXCL1 secretion by MSU stimulated BMDMs.

MSU crystals enhanced the expression of M1 markers; IL-1β, iNOS, and CCL2 (*p < 0.0001* for all comparisons; Figure 4A). We also observed a compensatory enhancement in M2 marker expression; IL-10 and arginase (*p < 0.0001*). Fingolimod treatment reduced the expression of M1 and M2 markers (*p < 0.001* for all comparisons). As a result of MSU crystal exposure, BMDMs enhanced the secretion of G-CSF, M-CSF and GM-CSF (*p < 0.05*; *p < 0.0001*; *p < 0.01*; Figure 4B), and fingolimod treatment reduced M-CSF secretion (*p < 0.05*). In addition, we detected elevated IL-1α, IL-1β, IL-6 and TNF-α levels in MSU stimulated BMDMs (*p < 0.001* for all comparisons; Figure 4C), and fingolimod treatment reduced these pro-inflammatory cytokines (*p < 0.05* for all comparisons). MSU crystal exposure induced the secretion of chemokines; CCL4 (*p < 0.01*), CCL5 (*p < 0.05*), CXCL1 (*p < 0.0001*) and CXCL2 (*p < 0.05*) by BMDMs (Figure 4D), and fingolimod treatment only reduced CXCL1 secretion (*p < 0.05*). Febuxostat treatment demonstrated a similar pattern of preferentially inhibiting cytokine release to chemokine release and its magnitude of inhibiting cytokine release was equivalent to that of fingolimod. Our data suggest that fingolimod and febuxostat effects were skewed towards suppression of pro-inflammatory cytokine production in macrophages, with a marginal impact on chemokine secretion by macrophages.

### Inflammatory CMs demonstrated enhanced XO expression and fingolimod treatment reduced the influx of inflammatory monocytes and neutrophils and enhanced anti-inflammatory NCMs in model acute gout

**FIGURE 5:**
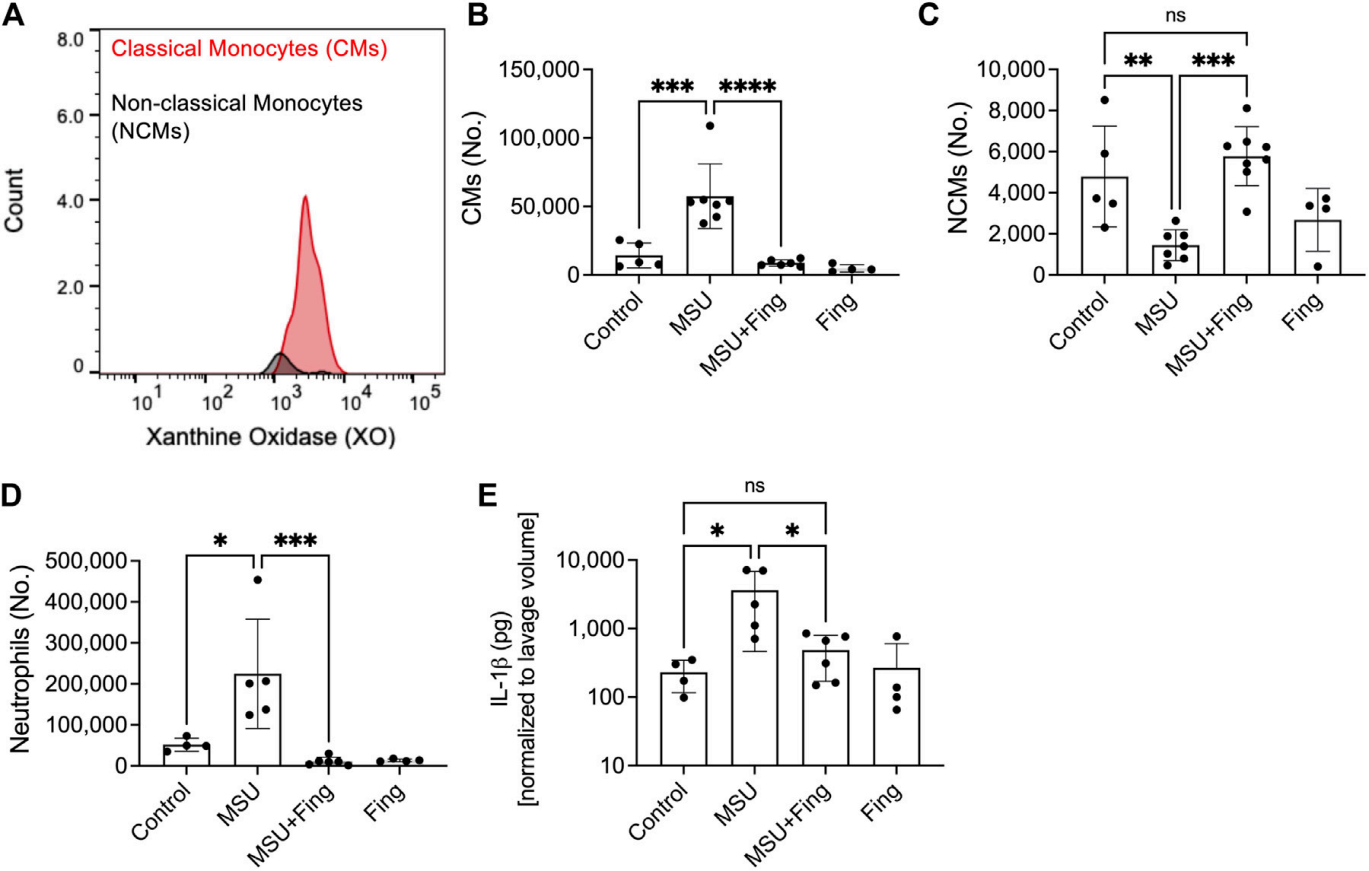
Regulation of inflammation in the murine peritoneal model of acute gout and *in vivo* efficacy of fingolimod. Recruitment of inflammatory classical monocytes (CMs), anti-inflammatory nonclassical monocytes (NCMs) and neutrophils in peritoneal lavages (PLs) of monosodium urate (MSU) crystal challenged mice. Peritoneal Lavaging was performed at 4 h following MSU crystals ± vehicle or fingolimod treatments. CMs, NCMs, and neutrophils were identified using surface markers by flow cytometry and PL cell populations of interest were determined using Precision Counting Beads. PL IL-1β levels were determined by ELISA. Xanthine oxidase (XO) expression in CMs and NCMs was determined by intracellular staining followed by flow cytometry. Data points represent individual mice. Statistical analysis was performed by ANOVA. ns, non-significant; *p < 0.05; **p < 0.01; ***p < 0.001; ****p < 0.0001. **(A)** XO was detected in CMs to a greater extent to NCMs. **(B)** Fingolimod treatment reduced CMs in peritoneal lavages of model acute gout. **(C)** Fingolimod treatment increased NCMs in peritoneal lavages of model acute gout. **(D)** Fingolimod treatment reduced neutrophils in peritoneal lavages of model acute gout. **(E)**. Fingolimod treatment reduced lavage IL-1β levels in model acute gout.

CMs demonstrated enhanced XO expression compared to NCMs (Figure 5A). MSU crystals resulted in higher lavage CMs (*p < 0.001*; Figure 5B), neutrophils (*p < 0.05*; Figure 5D) and lower lavage NCMs (*p < 0.01*; Figure 5C). In addition, MSU crystals increased lavage IL-1β levels (*p < 0.05*; Figure 5E). Fingolimod treatment reduced CMs (*p < 0.0001*), neutrophils (*p < 0.001*), IL-1β levels (*p < 0.05*) and increased NCMs (*p < 0.001*). Taken together, fingolimod demonstrated an anti-inflammatory effect in a model acute gout mediated by suppressing inflammatory immune cell recruitment and enhancing inflammation resolution mediated by enhanced NCM influx.

## Discussion

In this study, we have shown that PP2A activation suppressed XO expression and activity and blunted XO-derived ROS and UA generations in acute inflammation models of macrophages. The effect of fingolimod on XO activity was only demonstrated upon stimulation with MSU crystals or IL-1β with no significant inhibition of basal XO activity. This differential effect contrasts with febuxostat’s effect where the latter consistently reduced basal and MSU or IL-1β associated XO activity. The superior XO inhibitory effect of febuxostat corresponded with greater reductions in intracellular and secreted UA compared to fingolimod. The lack of a direct inhibitory effect by fingolimod argues that the drug’s effect was likely related to its ability to suppress IL-1β′s signaling pathway, resulting in reducing XO gene expression and by extension its activity. The signaling pathways of IL-1β and TLR2/TLR4 agonists (e.g., MSU crystals) have a common downstream effect of promoting the nuclear translocation of nuclear factor kappa b (NFκB) (Cohen, 2014), and we previously reported that PP2A inhibition increased NFκB nuclear translocation in urate crystal stimulated BMDMs (Bousoik et al., 2020). The contribution of soluble UA (sUA) to the overall inflammatory picture of gout is discrepant. sUA may have an independent pro-inflammatory effect on monocytes and macrophages, mediated by activation of the NLRP3 inflammasome (Crisan et al., 2016; Braga et al., 2017; Braga et al., 2021). However, it was also proposed that sUA may function as a negative regulator of monocyte activation by insoluble urate crystals (Ma et al., 2020). In our experience, sUA primed human THP-1 monocytes towards danger signals and enhanced IL-1β secretion (Qadri et al., 2021a). Specific to the UA generated by macrophages, XO-derived UA did not appear to contribute to NLRP3 inflammasome activation in macrophages (Ives et al., 2015). Arguably, systemic XO inhibition lowers serum UA levels and thus reduces the risk of urate crystal tissue deposition and acute gout flares, an effect that underpins the clinical efficacy of febuxostat (Becker et al., 2009).

A decline in macrophage PP2A activity was observed after urate crystal exposure and the direct enhancement of PP2A activity by fingolimod reduced IL-1β secretion. This observation is consistent with our prior observation that PP2A activity in human THP-1 monocytes was sensitive to combined TLR priming and urate crystal treatment (Qadri et al., 2021a). Interestingly, XO inhibition by febuxostat caused a reconstitution of PP2A activity in urate crystal treated macrophages. A direct PP2A activating effect as the mechanism of febuxostat may be ruled out as the drug alone, in contrast to fingolimod, did not increase PP2A activity above its basal level when incubated with macrophages. To identify whether PP2A inactivation was due to the accumulation of ROS in macrophages, which was reversible with febuxostat, we studied PP2A activity modulation following NAC treatment. NAC is an ROS scavenger that was previously shown to modulate macrophage function in response to LPS, specifically reducing ROS production and TNF-α release (Victor et al., 2003). NAC’s antioxidant effect was dependent on Nrf2 expression status in macrophages, where Nrf2 is a key transcriptional activator of antioxidant response in macrophages (Wang et al., 2019). Interestingly, both febuxostat and fingolimod treatments enhanced Nrf2 expression at the gene and protein levels, which might be significant in the context of reducing ROS burden, and their overall anti-inflammatory profile. In our model, NAC treatment lowered ROS burden and suppressed IL-1β secretion with an associated restoration of PP2A activity to its basal level and inhibition of XO activity. The recovery in PP2A activity appeared to mediate NAC’s anti-inflammatory efficacy as okadaic acid co-treatment attenuated the magnitude of IL-1β release with NAC. The impact of the redox status on PP2A activity is complex, cell context-dependent, and ROS’s effects span the structural and functional aspects of PP2A (Raman and Pervaiz, 2019). In primary neurons, ROS accumulation inactivated PP2A *via* tyrosine phosphorylation and leucine carboxy demethylation (Chen et al., 2009). In hematopoietic malignancies, ROS accumulation resulted in dissociation of the catalytic subunit of PP2A and PP2A activity suppression which resulted in inhibition of cell apoptosis (Nakahata and Morishita, 2014). Our findings are the first to demonstrate that ROS accumulation in macrophages inactivates PP2A as part of the mechanism of macrophage activation by urate crystals. Furthermore, we have also demonstrated that PP2A may play a significant role in modulating ROS generation and downstream effect in macrophages *via* regulation of XO activity, Nrf2 expression enhancement and caspase-1 activity reduction.

NLRP3 inflammasome activation is crucial to the processing of pro-IL-1β to mature IL-1β, mediated by caspase-1. We have observed that an NLRP3 inflammasome activator, nigericin, activated caspase-1 and induced IL-1β secretion by murine macrophages. In addition to generating ROS, nigericin also reduced PP2A activity. The specific contribution of PP2A to nigericin-induced NLRP3 inflammasome activation remains unclear. In one report, and using okadaic acid, NLRP3 inflammasome activation was reduced in response to nigericin (Martin et al., 2014), which suggests that PP2A activity is required for NLRP3 inflammasome activation (Martin et al., 2014). In that study, PP2A activity was not measured and thus it remains unclear what was nigericin’s effect on basal PP2A activity. In our experience, okadaic acid was shown to increase IL-1β release in urate crystal stimulated BMDMs, which suggests that PP2A negatively regulates NLRP3 inflammasome activation in response to urate crystals (Bousoik et al., 2020). The mechanism that underlines nigericin’s effect on PP2A activity level is unclear, but the generation of mitochondria-derived ROS is potentially implicated. Interestingly, neither fingolimod nor febuxostat reduced ROS generation linked to nigericin, in contrast to what was observed with NAC. To explain this observation, we posit that unlike NAC, fingolimod lacks intrinsic antioxidant effect and its reduction of XO-derived ROS production was due to its inhibitory effect on IL-1β signaling pathway and downstream XO expression and activity. In this light, febuxostat’s inability to reduce ROS in nigericin-stimulated BMDMs is also related to the source of ROS with the lack of involvement of XO. The reduction in caspase-1 activity by fingolimod was correlated with a reduction in IL-1β secretion, and our data suggest that fingolimod exerts a broad anti-inflammatory effect on macrophages regardless of the nature of the stimulus that causes NLRP3 inflammasome activation.

The anti-inflammatory profile of fingolimod encompasses both NLRP3 inflammasome dependent and independent effects as shown by its ability to inhibit the secretion of pro-inflammatory cytokines that do not require NLRP3 inflammasome processing e.g., IL-6 and TNF-α in addition to IL-1β. Fingolimod may also regulate M1 macrophage polarization as it reduced the expression of a key M1 marker, iNOS (Xue et al., 2018), while fingolimod’s effect on M-CSF secretion by macrophages is relevant to acute gout, given the cytokine’s role in priming resident macrophages in the setting of an acute flare (Liu et al., 2022). Fingolimod also regulated the trafficking of immune cells in an *in vivo* model of acute gout. CMs surged to the site of inflammation in our acute gout model. Owing to high expression levels of LyC6 and the pro-recruitment receptor CCR2, CMs extravasate into sites of inflammation where they differentiate into M1 macrophages (Martin et al., 2011; Cormican and Griffin, 2022). Fingolimod reduced CCR2+ CM trafficking into the site of inflammation as well as neutrophil ingress and secreted IL-1β. Combined, these effects would dampen inflammation in the tissue microenvironment. On the other hand, fingolimod increased the number of the anti-inflammatory NCMs, thereby aiding in the resolution of inflammation. NCMs are characterized by high CX3CR1 expression, which enables NCMs to play a potent and timely role in resolution of inflammation (Cormican and Griffin, 2022). Furthermore, NCMs differentiate into alternatively activated macrophages that function to promote repair and clearance of tissue debris (Cormican and Griffin, 2022). It is therefore reasonable to conclude that PP2A activation plays a dual role of suppressing inflammatory immune cell trafficking to the site of inflammation while simultaneously resolving inflammation, mediated by enhanced NCM ingress. In our *in vitro* experiments, we utilized febuxostat at 200 μM, the concentration that was reportedly used to demonstrate that XO-derived ROS activated NLRP3 inflammasome and mediated IL-1β release (Ives et al., 2015). At concentrations up to 100 μM, febuxostat had no off-target effects on other enzymes of the purine metabolism pathway (Takano et al., 2005). It is therefore plausible to expect that selectivity of febuxostat for XO remains at 200 μM. Regardless, our data support that PP2A activation can regulate XO activity in macrophages and that inactivation of PP2A contributes to macrophage stimulation by urate crystals. Lastly, another limitation of our study is that we did not compare the *in vivo* efficacy of fingolimod to that of febuxostat.

In conclusion, we have shown that PP2A is an important target in urate crystal induced macrophage activation and downstream pro-inflammatory cytokine secretions. PP2A is inactivated by XO-derived ROS upon urate crystal stimulation of macrophages. Furthermore, the efficacy of a broad antioxidant such as NAC was mediated by PP2A activity restoration, and associated inhibition of XO activity. Fingolimod reduced XO expression and by extension inhibited XO activity, with an effect size comparable to febuxostat. Fingolimod phosphate was also efficacious in blunting the acute phase of inflammation and promoting its resolution an *in vivo* murine acute gout model. PP2A activation is thus a promising new therapeutic strategy for acute gout flares.

## Data Availability

The raw data supporting the conclusions of this article will be made available by the authors, without undue reservation.
